# Comparative Analysis of Composite Mortality Prediction Scores in Intensive Care Burn Patients

**DOI:** 10.3390/ijerph191912321

**Published:** 2022-09-28

**Authors:** Doha Obed, Mustafa Salim, Nadjib Dastagir, Samuel Knoedler, Khaled Dastagir, Adriana C. Panayi, Peter M. Vogt

**Affiliations:** 1 Department of Plastic, Aesthetic, Hand and Reconstructive Surgery, Hannover Medical School, 30625 Hannover, Germany; 2 Department of Human Genetics, Hannover Medical School, 30625 Hannover, Germany; 3 Department for Plastic Surgery and Hand Surgery, Klinikum Rechts der Isar, Technical University of Munich, 81675 Munich, Germany; 4 Division of Plastic Surgery, Department of Surgery, Brigham and Women’s Hospital, Harvard Medical School, Boston, MA 02215, USA

**Keywords:** burn intensive care, ABSI score, BAUX, BOBI, BUMP score, burn mortality, burn survival

## Abstract

Multiple outcome scoring models have been used in predicting mortality in burn patients. In this study, we compared the accuracy of five established models in predicting outcomes in burn patients admitted to the intensive care unit and assessed risk factors associated with mortality. Intensive care burn patients admitted between March 2007 and December 2020 with total body surface area (TBSA) affected ≥ 10% were analyzed. Multivariate analysis was conducted to examine variables associated with mortality. The ABSI, Ryan, BOBI, revised Baux and BUMP scores were analyzed by receiver operating characteristics. A total of 617 patients were included. Morality was 14.4%, with non-survivors being significantly older, male, and having experienced domestic burns. Multivariate analysis identified age, TBSA, full-thickness burns and renal insufficiency as independent mortality predictors. The BUMP score presented the highest mortality prognostication rate, followed by ABSI, revised Baux, BOBI and Ryan scores. BUMP, ABSI and revised Baux scores displayed AUC values exceeding 90%, indicating excellent prognostic capabilities. The BUMP score showed the highest accuracy of predicting mortality in intensive care burn patients and outperformed the most commonly used ABSI score in our cohort. The older models displayed adequate predictive performance and accuracy compared with the newest model.

## 1. Introduction

Burn injuries present a major cause of mortality and morbidity worldwide and continue to be among the costliest traumatic wounds, due to extensive hospitalization and rehabilitation times and expensive post-injury treatment [1,2]. Several burn prevention strategies, e.g., the use of smoke detectors, fire-resistant electrical wires, flameproof shielding gear and standard operation procedure protocols in work facilities, have affected a reduction of burn injuries in the last decades. Nonetheless, burn-related mortality still remains high for patients with severe burns [3,4].

In light of the shift towards outcome-based performance measures, mortality prediction is increasingly becoming an important aspect of clinical burn care. Efforts to provide objective approximations of the risk of death after severe burn injuries have an extensive history in burn care research. In recent years, several prognostic models, scores and indexes have been introduced as useful tools for standardizing and improving comparability of quality of burn care and research and to assist in identifying patients at risk who may require additional attention and resources. In order to provide a reliable severity-adjusted outcome monitoring of thermal injuries, these models have to deliver accurate predictions. However, their accuracy and applicability have been questioned conflictingly in the literature [3,4,5].

Well-designed comparisons of these models are sparsely documented in the burn literature [6]. Besides, recent improvements in burn and intensive care, particularly in infection prevention, wound care, fluid management and renal replacement therapy, have aided in reducing burn-related mortality [7]. These advancements may have limited the predictive strength of earlier-introduced prognostic tools. We examined the accuracy and validity of previously proposed and validated prognostic models in a large single-center cohort of intensive care burn patients to examine the most suitable prognostic score and to advance our understanding in the care of burn patients.

## 2. Methods

### 2.1. Study Design and Data Extraction

The data for this retrospective study were collected from the records of patients admitted to the burn intensive care unit (ICU) at Hannover Medical School from March 2007 to December 2020. This center is one of the major burn centers in Northern Germany with 6 ICU beds.

The complete records of 1379 patients admitted to the burn ICU were reviewed. Patients over the age of 16 years and with total body surface area (TBSA) affected ≥10% were included. “Do-Not-Resuscitate” status has not been considered as an inclusion criterion. In total, 617 patients have been included in the final analysis. Demographic and clinical parameters were collected including age, gender, inhalation injury, burn site and severity, TBSA, comorbidities, as well as location and cause of burn accident at the time of the burn injury. Data on in-hospital mortality, length of stay and mechanical ventilation were also collected. This research was conducted according to the principles of the Declaration of Helsinki. It was exempt from approval by the Ethics Committee of Hannover Medical School due to its retrospective nature.

We proceeded to assess and compare the following five established and validated burn mortality prediction models (Table 1), as described in the primary publications (Supplementary Table S1):

1. The abbreviated burn severity index (ABSI) published by Tobiasen et al. in 1982. This remains one of the most commonly applied mortality prediction scores in burn care [8]. It is based on the sum of scaled values for age, TBSA, sex, inhalation injury and full-thickness burns [9].
2. The Ryan score published by Ryan et al. in 1998. It is based on the presence of scaled risk factors using the parameters age, TBSA and inhalation injury [10].
3. The Belgian Outcome in Burn Injury (BOBI) score published by the Belgian Outcome in Burn Injury Study Group in 2009. This model is based on the parameters age, TBSA and inhalation injury [11].
4. The revised Baux score as published by Osler et al. in 2010. This relies on the risk factors age, TBSA and inhalation injury [12].
5. The Burn Mortality Prediction (BUMP) score published by Bagheri et al. in 2022 is based on the parameters age, TBSA, presence of inhalation injury, full-thickness burns, and the circumstances and risk factors present [13].

### 2.2. Statistical Analysis

Data were collected and organized using Microsoft Excel (Version 16, Microsoft Corporation, Redmond, WA, USA). Data analysis and descriptive statistics were performed using GraphPad Prism 9 (GraphPad Software Inc., San Diego, CA, USA) and Microsoft Excel. A binomial test was used for comparative categorical analyses. A *t*-test was used to compare quantitative variables. *p*-values < 0.05 were considered significant. Variables with a *p*-value < 0.1 in the univariate logistic analysis were included in the subsequent multivariate logistic regression analysis. Variable multicollinearity was examined by evaluating the variance inflation factor (VIF). Variables that showed a VIF > 4.0 were excluded from the final analysis. Receiver operating characteristic (ROC) curves were plotted in order to assess the performance of included scores in predicting mortality in the present study population, as evidenced by the area under the curve (AUC). The subsequent cutoff point was determined using the Youden index. The corresponding sensitivity and specificity of each prediction model were reported. Goodness of fit of the logistic regression models was evaluated using the log likelihood ratio test (LRT).

## 3. Results

During the 14-year study period, 617 ICU burn patients with TBSA affected ≥10% were admitted to Hannover Medical School, Germany; 71.5% and 28.5% were male and female, respectively (male to female ratio 2.5:1). On average, patients were 49 years old. The most common burn etiology was flame and contact burn (55.3%), followed by explosion and deflagration (22.7%), scalding (18.6%), chemical (1.8%) and electrical burns (1.8%). The most frequently affected burn sites included the arms (70.7%), the face/neck/scalp area (58.3%), legs (53.6%), hands (50.7%) and thorax (47.2%). Of note, 42.8% of the patients sustained full-thickness burns, and the average TBSA was 23.2%.

Most burn injuries occurred domestically (55.3%), whereas burns during recreational activities (23.7%) and work-related activities (15.6%) were proportionally less frequent. 15.9% of cases presented with inhalation injuries. The clinical characteristics, as well as outcome parameters are summarized in Table 2.

In total, we noted a mortality rate of 14.4% with 89 deaths among the cohort. Non-survivors were significantly older (61.9% vs. 46.7%, *p* < 0.001), mostly male and more frequently experienced domestic burns (70% vs. 52.7%, *p* < 0.001). In addition, 42.7% of the non-survivors were over the age of 64. They presented with significantly higher burn severity, depicted by greater TBSA (43.5% vs. 19.8%, *p* < 0.001), higher rates of inhalation injury (21.3% vs. 15%, *p* <0.016) and full-thickness burns (88.8% vs. 35%, *p* < 0.001). Non-survivors significantly suffered more frequently from concomitant comorbidities including coronary artery disease, chronic obstructive pulmonary disease and heart insufficiency. This was particularly the case for history of arrhythmia (43.8% vs. 9.5%, *p* < 0.001) and renal insufficiency (47.2% vs. 3.2%, *p* < 0.001). While there was an increased need for mechanical ventilation (52.2% vs. 33.9%, *p* < 0.001) and prolonged ventilation times noted (167 h vs. 58 h, *p* < 0.001), non-survivors showed a decreased total length of hospital stay (LOS) compared to survivors (16 days vs. 27 days, *p* < 0.001). Non-survivors were more frequently associated with a history of attempted suicide (12.2% vs. 3.8%, *p* < 0.001).

**Figure 1:**
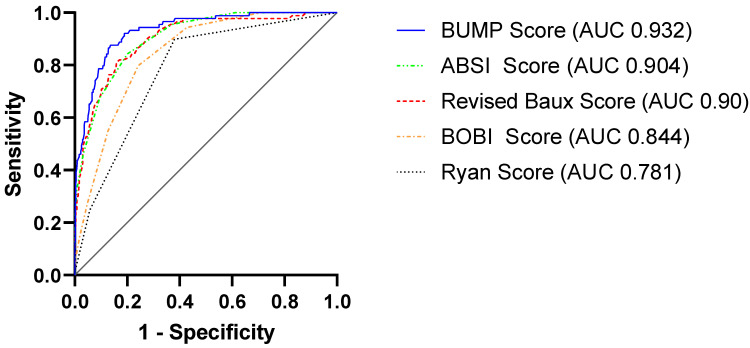
Receiver operating characteristic (ROC) and area under the curve (AUC) displaying the predictive value of the assessed mortality prediction scores.

Multivariate analysis identified age, TBSA, full-thickness burns and renal insufficiency as independent parameters associated with in-hospital mortality (see Table 3). Consequently, we conducted a comparison of the following prognostic burn scores: ABSI, Ryan, BOBI, revised Baux and BUMP (see Figure 1). All assessed burn mortality scores were greater in non-survivors, indicating their prognostic capability with regard to mortality after severe burns. Predictive ability was assessed by area under the curve (AUC), which proved to be adequate (see Table 4). The BUMP score had the highest AUC value at 0.932 (95% CI: (0.906–0.958)). The ABSI score had the second-highest AUC value at 0.904 (95% CI: 0.875–0.934), followed by the revised Baux score at 0.9 (95% CI: 0.866–0.935) and the BOBI score at 0.844 (95% CI: 0.807–0.881). The AUC value of the Ryan score was 0.781 (95% CI: 0.734–0.828). Accordingly, it has ranked last with regard to AUC among the examined prognostic models.

## 4. Discussion

In this study, we applied five of the most commonly utilized burn injury scoring models and evaluated their accuracy to predict in-hospital mortality. Our results suggest that the recently introduced BUMP score fitted the data retrieved from our burn unit and that it delivered excellent discrimination properties compared to the pre-existing prognostic models given the AUC value of 0.93. Despite a lower AUC value when compared to the BUMP score, our results similarly support the prognostic ability of the ABSI score to estimate mortality in our unit with superior prognostic value, when compared to previously published AUC values [14,15,16].

Although based on different population profiles, our results and the original publications of the ABSI and BUMP scores both acknowledge significant factors that display distinct influence on mortality in multivariate analysis (age, TBSA-affected and full-thickness burns). Particularly, age and TBSA have been considered in almost all predictive models presented in the burn literature. TBSA is a strong predictor of mortality and remains one of the most objective estimates of burn severity. The influence of age on burn outcome becomes apparent when considering its role in the pathophysiologic processes: recovery from burn injury, regardless of severity, is impaired in the presence of chronic illness and physiologic changes in the elderly [17,18]. Additionally, reduced immune function and altered metabolic responses, including delayed hypermetabolic response, increased hyperglycemic and hyperlipidemic response, and inverse inflammatory response, are known to negatively impact wound healing capabilities, which are associated with aging [19].

We also found full-thickness burns to be significantly associated with mortality in the multivariate analysis. This is in agreement with multiple recently published studies [20,21,22]; however, there are also conflicting data in the literature [23,24]. It is worth noting that estimates of burn depth on admission remain at best subjective and susceptible to interobserver variability. Burn depth is also prone to evolve during in-hospital stay, which may account for the fact that accurate assessment of burn depth remains possible in only 64 to 76% of cases, even for experienced burn surgeons [25]. The fact that we observed an increased mortality in patients with full-thickness burns implies that surgical management may have a negative influence on survival probability. Therefore, particular care has to be taken when planning for surgical interventions. Uncompromising strategies including early debridement and skin grafting may have beneficial effects on patients’ outcome; however, adverse results may occur in critically ill and unstable patients. Future research will have to address and further elucidate under which circumstances incorporating the concept of damage control surgery may prove to be favorable and when to opt for safe definitive surgery [26].

Despite including these significant risk factors, a shortfall of the ABSI score and the subsequent models is a lack of incorporation of comorbidities and burn injury circumstances, which may result in an underestimation of mortality [27]. Integration of these variables in the BUMP score has addressed this limitation of prior burn mortality scores and established its superiority over several pre-existing burn-specific models.

Accordingly, we found that non-survivors suffered more frequently from concomitant comorbidities including coronary artery disease, chronic obstructive pulmonary disease, heart insufficiency, arrhythmia, and renal insufficiency. Fittingly, burn patients with pre-existing or new-onset renal insufficiency have been shown to be associated with a significantly higher occurrence of in-hospital mortality compared to burn patients without a history of renal disease [28,29]. Previous studies have found mortality rates of up to 34.9% for burn patients with new-onset kidney disease and of up to 65.5% for burn patients who required renal replacement therapy [30,31]. Multivariate analysis identified renal insufficiency as an independent parameter associated with in-hospital mortality in our cohort. Approximately 70% of our patients diagnosed with renal insufficiency have expired during the study period, rendering it a considerable covariate when estimating survival probability. However, future studies will be imperative to stratify risk assessment for various stages and forms of renal insufficiency. Susceptibility to renal disease following burn injuries is particularly pronounced during two periods: firstly, immediately following burn shock, which is marked by pre-renal insufficiency due to hypovolemia, elevated systemic vascular resistance and myocardial suppression; secondly, it can result from circulatory inflammatory mediators and gram-negative sepsis in later stages [32,33]. Consequently, monitoring of renal function remains integral throughout all periods of in-hospital treatment, given its considerable effect on mortality.

Severe burn injuries to date result in acute phase reactions, which may be followed by an extensive inflammatory systemic response and multiple organ dysfunction [34,35,36]. Outcomes following burn injury, and particularly, mortality, are accordingly subject to patients’ physiological reserves, which decline with the presence of comorbidities and increasing age. In the clinical practice of burn care, mortality prediction is largely based on the use of burn severity scores. Prognostic models used to calculate expected mortality following burn injuries are mainly based on burn injury-specific parameters with a special focus on burn severity and can sometimes incorporate comorbidities and burn injury circumstances. The ideal burn mortality model should consequently combine a high degree of accuracy with simplicity of use.

Despite the vast number of burn mortality prediction scores, accurate prediction remains variable and dependent on the examined population and its region [37,38,39,40]. This may imply the heterogeneity of burn patients and differences in regional standards of care, which may affect burn injury outcome and, consequently, mortality prediction. To date, there is no universal model established as superior for burn mortality prediction. In fact, the existing scores and models represent differently weighted accounts of similar variables. Therefore, external validation of these scores remains essential to identify the most suitable prognostic model for regional clinical application.

Taken together, the integration of comorbidities and burn injury circumstances in the BUMP score has led to ameliorated accuracy and predictive capabilities in burn mortality prediction in our cohort. However, we acknowledge that the degree of complexity of the prognostic model determines its usefulness in both clinical and research applications. Models that are based on simple calculations and easily accessible variables, e.g., the ABSI score, are suitable for initial risk assessment and bedside triage. Despite higher accuracy, the BUMP score may be less suitable for initial triage in light of unclear comorbidities and burn injury circumstances.

It should be noted that burn mortality scores are not a replacement of sound clinical judgment and should not be independently used to guide allocation of care. Rather, they present a prediction of outcomes after provision of burn care and a potential tool for monitoring the quality of burn center performance and the progress following the implementation of therapeutic modalities and concepts. Consequently, as treatment concepts transform and evolve, so should these prognostic models.

## 5. Limitations and Future Work

The presented results should be interpreted with consideration of the limitations of our study. Our data are based on a cohort from a single center that involves patients in the northern region of Germany. As such, the study suffers from geographical bias and our conclusions may not be generalizable. Future, multi-institutional studies could help establish the external validity of our results. Also, our results are based on observations of burn patients presenting with injuries affecting ≥10% of TBSA. This may have introduced a significant bias given that patients with <10% TBSA may have also had severe comorbidities and non-comparable outcomes. Furthermore, the retrospective nature of this study is associated with inherent biases including a higher susceptibility to confounding and selection bias. A large prospective study comparing the five main predictive models would reduce such biases. Additionally, “Do-Not-Resuscitate” status has not been included in the analysis as a covariate, which may have affected mortality performance.

## 6. Conclusions

Our results identify the BUMP score as having superior ability to predict mortality compared to previously published prognostic mortality models, when applied to a cohort of burn patients admitted to a large academic hospital’s burn ICU. The ABSI, BOBI and revised Baux scores were, however, statistically accurate predictors. Age, TBSA, full-thickness burns and renal insufficiency were identified as independent risk factors for burn-related in-hospital mortality in our cohort.

## Figures and Tables

**Table 1:** Mortality prediction scores.

Prognostic Score	Year	Parameters Used						
		Age	TBSA	IHT	Full-Thickness Burn	Sex	Comorbidities	Circumstances
ABSI	1982	x	x	x	x	x		
Ryan	1998	x	x	x				
BOBI	2009	x	x	x				
Revised Baux	2010	x	x	x				
BUMP	2022	x	x	x	x		x	x

TBSA: total body surface area; IHT: inhalation trauma; ABSI: Abbreviated Burn Severity Index; BOBI: Belgian Outcome in Burn Injury; BUMP: burn mortality prediction.

**Table 2:** Clinical characteristics and outcomes.

Variable	Total (*n* = 617)	Survivors (*n* = 528)	Non-Survivors (*n* = 89)	*p*-Value
Age (years) (mean ± SD)	48.9 ± 19	46.7 ± 18.3	61.9 ± 18.7	<0.001
TBSA (%) (mean ± SD)	23.2 ± 16	19.8 ± 10.6	43.5 ± 13.9	<0.001
Male Gender, *n* (%)	441 (71.5)	379 (71.8)	63 (70.8)	0.06
Age Group, *n* (%)				
14–24	74 (12.0)	70 (13.3)	4 (4.5)	0.139
25–44	189 (30.6)	177 (33.5)	12 (13.5)	0.029
45–64	225 (36.5)	190 (36.0)	35 (39.3)	0.057
≥65	129 (20.9)	91 (17.2)	38 (42.7)	<0.001
TBSA, *n* (%)				
10–19.9	329 (53.3)	315 (59.7)	14 (15.7)	<0.001
20–29.9	140 (22.7)	120 (22.7)	20 (22.5)	0.283
≥30	148 (24.0)	93 (17.6)	55 (61.8)	<0.001
Inhalational Injury, *n* (%)	98 (15.9)	79 (15.0)	19 (21.3)	0.016
Full-Thickness Burns, *n* (%)	264 (42.8)	185 (35.0)	79 (88.8)	<0.001
Comorbidities, *n* (%)				
Hypertension	129 (20.9)	110 (20.8)	19 (21.3)	0.211
Diabetes	53 (8.6)	43 (8.1)	10 (11.2)	0.084
Peripheral Arterial Disease	9 (1.5)	6 (1.1)	3 (3.4)	0.072
Coronary artery Disease	30 (4.9)	19 (3.6)	11 (12.4)	<0.001
Chronic Obstructive Pulmonary Disease	19 (3.1)	13 (2.5)	6 (6.7)	0.015
Arrhythmia	89 (14.4)	50 (9.5)	39 (43.8)	<0.001
Heart Insufficiency	16 (2.6)	9 (1.7)	7 (7.9)	0.001
Renal Insufficiency	59 (9.6)	17 (3.2)	42 (47.2)	<0.001
Burn Etiology, *n* (%)				
Flame/Contact	341 (55.3)	272 (51.5)	69 (77.5)	<0.001
Scalding	115 (18.6)	107 (20.3)	8 (9)	0.182
Explosion/Deflagration	140 (22.7)	130 (24.6)	10 (11.2)	0.141
Chemical	11 (1.8)	10 (1.9)	1 (1.1)	0.999
Electricity	11 (1.8)	10 (1.9)	1 (1.1)	0.999
Body Area, *n* (%)				
Face/Neck/Scalp	360 (58.3)	301 (57.0)	59 (66.3)	0.004
Arms	436 (70.7)	363 (68.8)	73 (82)	<0.001
Hands	313 (50.7)	265 (50.2)	48 (53.9)	0.032
Legs	331 (53.6)	270 (51.1)	61 (68.5)	<0.001
Feet	87 (14.1)	65 (12.3)	22 (24.7)	<0.001
Thorax	291 (47.2)	233 (44.1)	58 (65.2)	<0.001
Abdomen	157 (25.4)	126 (23.9)	31 (34.4)	0.002
Back/Flanks	199 (32.3)	148 (28.0)	51 (56.7)	<0.001
Genital area	51 (8.3)	34 (6.4)	17 (18.9)	<0.001
Place of Burn Incident, *n* (%)				
Home	341 (55.3)	278 (52.7)	63 (70)	<0.001
Recreational	146 (23.7)	134 (25.4)	12 (13.3)	0.295
Workplace	96 (15.6)	88 (16.7)	8 (8.9)	0.423
Other	34 (5.5)	28 (5.3)	6 (6.7)	0.27
Traffic Accident, *n* (%)	5 (0.8)	3 (0.6)	2 (2.2)	0.101
Suicide Attempt, *n* (%)	31 (5.0)	20 (3.8)	11 (12.2)	<0.001
LOS (days) (mean ± SD)	25.7 ± 23.3	27.4 ± 24.2	16 ± 3.7	<0.001
LOS on ICU (days) (mean ± SD)	15.9 ± 20.2	15.9 ± 21.1	15.8 ± 13.7	0.966
Surgical Intervention Rate (mean ± SD)	3.5 ± 3.7	3.4 ± 3.8	3.9 ± 2.9	0.237
Mechanical Ventilation, *n* (%)	227 (36.8)	180 (33.9)	47 (52.2)	<0.001
Mechanical Ventilation (hours) (mean ± SD)	73.6 ± 253.3	57.8 ± 245.3	166.6 ± 276.8	<0.001

TBSA: total body surface area; SD: standard deviation; LOS: length of hospital stay; ICU: intensive care unit.

**Table 3:** Multivariate analysis of variables.

Variable	*p*-Value	OR	CI (95%)
Age	<0.001	1.053	1.029–1.079
TBSA	<0.001	1.095	1.069–1.127
Full-Thickness Burns	<0.001	4.78	2.043–12.27
Coronary Artery Disease	0.451	1.541	0.4850–4.675
Arrhythmia	0.198	1.669	0.7575–3.621
Heart Insufficiency	0.354	1.854	0.4948–6.911
Renal Insufficiency	<0.001	9.042	4.000–21.33
Home	0.777	0.799	0.189–4.455
Recreational	0.124	0.255	0.0472–1.616
Workplace	0.22	0.28	0.0357–2.235
Suicide Attempt	0.6	0.66	0.128–2.902
Mechanical Ventilation	0.934	1.031	0.501–2.097
Inhalation Injury	0.058	2.293	0.961–5.402

TBSA: total body surface area; OR: odds ratio; CI: confidence interval.

**Table 4:** Comparative analysis of prognostic models.

Burn Mortality Score	Total (*n* = 619)	Survivors (*n* = 530)	Non-Survivors (*n* = 89)	Sensitivity	Specificity	AUC (95% CI)
ABSI (mean ± SD)	6.6 ± 2.2	6.1 ± 1.7	9.7 ± 2.2	0.843	0.629	0.904 (0.875–0.934)
Ryan (mean ± SD)	0.5 ± 0.7	0.4 ± 0.6	1.2 ± 0.6	0.899	0.619	0.781 (0.734–0.828)
BOBI (mean ± SD)	1.9 ± 1.7	1.6 ± 1.5	3.8 ± 1.6	0.798	0.758	0.844 (0.807–0.881)
Revised Baux (mean ± SD)	74.9 ± 25.7	69.2 ± 21.2	108.9 ± 23.6	0.809	0.841	0.900 (0.866–0.935)
BUMP (mean ± SD)	−3.1 ± 2.1	−3.6 ± 1.6	0.01 ± 1.8	0.876	0.864	0.932 (0.906–0.958)

TBSA: total body surface area; ABSI: abbreviated burn severity index; BOBI: Belgian outcome in burn injury; BUMP: burn mortality prediction; SD: standard deviation; CI: confidence interval; AUC: area under the curve.

## Data Availability

The data presented in this study are available on request from the corresponding author.

## Supplementary Materials

The following supporting information can be downloaded at: https://www.mdpi.com/article/10.3390/ijerph191912321/s1, Table S1: Mortality prediction scores and their mathematical equations.
