# Supplementary material for: Comparative Analysis of Composite Mortality Prediction Scores in Intensive Care Burn Patients
Source: Int J Environ Res Public Health. 2022 Sep 28;19(19):12321. doi: 10.3390/ijerph191912321 (PMC9564531; doi:10.3390/ijerph191912321)
Supplement: Supplementary file 1 [file ijerph-19-12321-s001.zip › ijerph-1917587-supplementary.pdf]

Supplemental Table S1. Mortality prediction scores and their mathematical equations.

Prognostic Score	Mathematical equation
ABSI	Age (0–20: 1;21–40: 2;41–60: 3;61–80: 4; 81–100: 5) + TBSA (%) (0–10: 1; 11–20: 2; 21–30: 3; 31–40: 4; 41–50: 5; 51–60: 6; 61–70: 7; 71–80: 8; 81–90: 9; 91–100: 10) + IHT (yes: 1; no: 0) + Full Thickness Burn (yes: 1; no: 0) + Sex (female: 1; male: 0)
Ryan	Logit = −5.89 + 2.58x; x= sum of risk factors (age > 60: 1; TBSA (%) >40: 1; IHT: 1)
BOBI	Age (<50: 0; 50–64: 1; 65–79: 2; ≥ 80: 3) + TBSA (%) (<20: 0; 20–39: 1; 40–59: 2; 60–79: 3; ≥80: 4) + IHT (yes: 3; no: 0)
Revised Baux	Age + TBSA (%) + (17 * IHT)
BUMP	−6.7 + (Age (40–49: 0.9; 50–59: 1.6; 60–69: 1.9; 70–79: 2.7; ≥ 80: 3.6)) + (TBSA (%) x 0.08) + (IHT (yes: 1; no: 0)) + (Full Thickness Burn (yes: 0.5; no: 0)) + (Number of Risk Factors: (1: 0.2; 2: 0.8; 3: 1.2; ≥4: 1.6)) + (Circumstances: (work-related: −0.5; traffic: 1.7; susp. suicide: 0.8))

TBSA: Total body surface area; IHT: Inhalation trauma; ABSI: Abbreviated burn severity index; BOBI: Belgian outcome in burn injury; BUMP: Burn mortality prediction.
